# Eliminating nonuniform geometric effects for long-term stable electrochemical extraction of high-purity titanium

**DOI:** 10.1126/sciadv.ads7083

**Published:** 2025-03-21

**Authors:** Zhiyuan Li, Shuqiang Jiao, Jun Zhu, Shijie Li, Zhaoliang Qu, Xiaodong Chen, Qi Wang, Shanyan Huang, Hao-Sen Chen, Wei-Li Song, Yingjun Liu, Dongbai Sun, Hongmin Zhu, Daining Fang

**Affiliations:** ^1^ Institute of Advanced Structure Technology, Beijing Institute of Technology, Beijing 100081, P. R. China.; ^2^ State Key Laboratory of Advanced Metallurgy, University of Science and Technology Beijing, Beijing 100083, P. R. China.; ^3^ State Key Laboratory of Advanced Processing and Recycling of Non-ferrous Metal. Lanzhou University of Technology, Lanzhou 730050, P.R. China.; ^4^ Ningxia Deyun Chuangrun Titanium Industry Co. Ltd., Ningxia Province 753000, P. R. China.; ^5^ School of Materials Science and Engineering, Sun Yat-sen University, Guangzhou 510006, P.R. China.; ^6^ China & Southern Marine Science and Engineering Guangdong Laboratory, Zhuhai 519082, P. R. China.; ^7^ Department of Materials Science & Metallurgy, University of Cambridge, 27 Charles Babbage Road, Cambridge CB30FS, UK.; ^8^ Tohoku University, 6-6-02, Aramaki-Aza-Aoba, Aobo-ku, Sendai 980-8579, Japan.

## Abstract

Electrorefining of low-grade titanium is one of the strategies for achieving high-purity titanium. However, the presence of nonuniform geometric effects would be induced to impact the nonuniform geometric distribution of overpotential, leading to impurity dissolution and nonuniform Ti deposition. Here, in situ high-temperature characterizations on the molten salt electrorefining process are applied to establish an anodic dissolution principle for quantitatively evaluating nonuniform geometric effects of electrode. For eliminating the nonuniform geometric effects, coaxial anode-cathode configurations are designed to promote the nonuniform anodic dissolution and nonuniform cathodic deposition. Consequently, the geometric uniformity of titanium products on the cathodes is substantially enhanced, and thus, long-term stable electrorefining process (~12 hours, ~330% increment compared to the electrode of reference configuration) and highly purified titanium products (99.2%) are achieved.

## Teaser

Promoting uniformity of electrode geometry is effective for long-term preparing high-purity Ti.

## INTRODUCTION

High-purity titanium is widely used in high-performance integrated circuits (*1*–*6*), electronics (*7*, *8*), and aerospace industry (*9*, *10*). Now, the primary technique for industrial production of high-purity titanium is thermal reduction (*11*) (known as Kroll process), along with attempted methods in iodization (*12*, *13*), electron beam melting (*14*), and zone refining (*15*). The products obtained by the industrial thermal reduction method (known as a long process flow) should be further purified by additional impurification, leading to promoted processing cost and decreased production efficiency. Using iodization method and zone melting method, on other hand, there is also a challenge in substantially promoting production efficiency (*16*), thereby limiting potential for large-scale application. In addition, electrorefining using molten salt electrolysis with short process flow, high production efficiency, and scalable capability is considered as a notable approach for depositing high-purity titanium (*17*, *18*).

In the electrorefining process, there are some essential challenges in stably producing high-purity titanium owing to three critical issues: (i) chemical composition variation in molten salt (*19*) (i.e., change in electrochemical equilibrium), (ii) variable electrochemical kinetics under a certain processing parameter (*20*, *21*) (i.e., impact in crystalline growth on cathode), and (iii) nonuniform geometric distribution of current density and overpotential on both anodes/cathodes (i.e., induced impurity dissolution and deposition). To address the issues (i) and (ii), introduction of additives for changing electrolyte compositions (*22*, *23*) and manipulation of the electrorefining processing parameters (*24*, *25*) have been proved to improve the stability of the electrolytic processes. As a common behavior of the geometric changes on both anodes and cathodes, however, three types of nonuniform geometric effects (i.e., distance effects, edge effect, and bottom effect) would be generally induced to substantially affect the nonuniform geometric distribution of current density and overpotential. These nonuniform geometric effects would essentially lead to impurity dissolution and nonuniform Ti deposition in the anodic and cathodic processes, respectively. Unfortunately, there is a technical bottleneck to quantitatively acquire and evaluate these nonuniform geometric effects, and thus, rational geometric design on anode-cathode configurations for long-term stable Ti electrorefining process is still a great challenge.

To overcome the challenge, here, we used the in situ four-dimensional (4D) (3D space and additional time dimension) characterizations to quantitatively study the geometric electrode changes during electrochemical process of titanium. By establishing a dissolution principle of analyzing soluble anodes, threshold values of the time-dependent applied current density and time-dependent applied overpotential could be used to evaluate the nonuniform geometric effects of anodic process and cathodic Ti deposition. For substantially eliminating all these three types of geometric effects, a variety of coaxial anode-cathode configurations have been designed to promote the nonuniform dissolution of anode and nonuniform deposition of cathode. Experiments has been carried out to verify the anode-cathode configurations for Ti electrorefining, which suggests that the geometric uniformity and purity of titanium products has been promoted from 2.28 to 4.17 (increment: 83%) and from 96.6 to 99.2 wt % (increment: 2.7%), respectively. The as-established principle upon the 4D analysis method and anode-cathode configuration would be widely extended in electrorefining metals, aiming to achieving high-purity metals in a scalable strategy.

## RESULTS

### Anodic dissolution principle based on 4D analysis of Ti dissolution behaviors

**Fig. 1.**
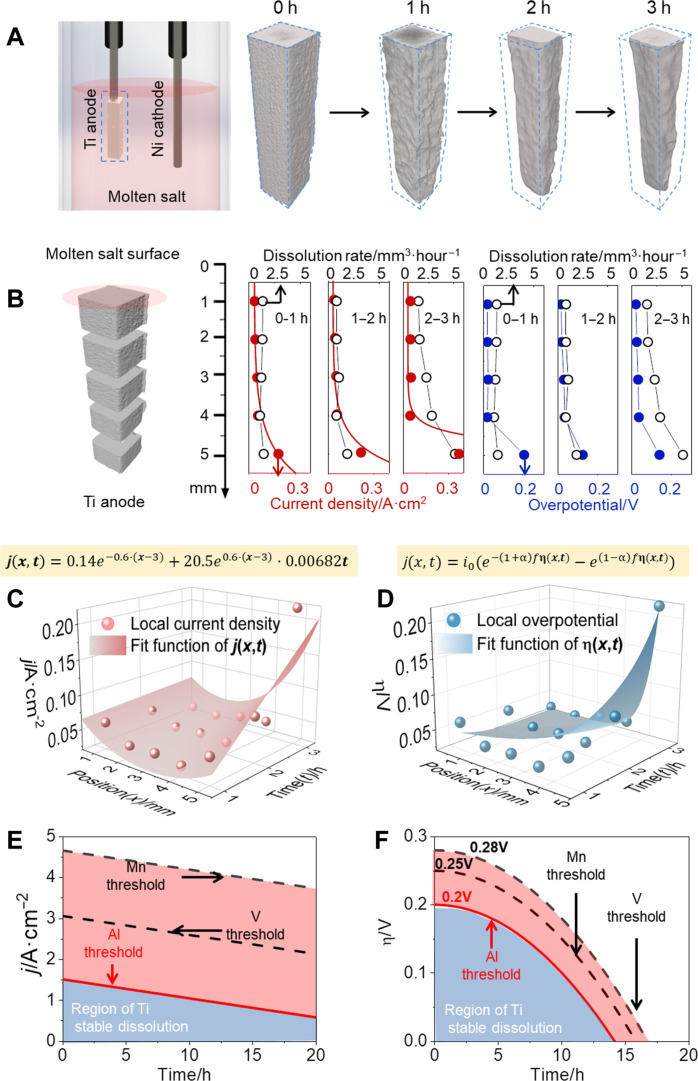
Anodic dissolution principle based on 4D analysis of Ti dissolution behaviors. (**A**) Schematics of electrolysis cell and 3D reconstructed images of the Ti anode at different electrolysis time ranges. (**B**) Schematic of electrode segmentation and current density and overpotential in different electrode regions. (**C**) Functional relationship and fitting of current density with electrode height and electrolytic time. (**D**) Functional relationship and fitting of overpotential with electrode height and electrolytic time. (**E**) Correlation between initial current density and the duration of stable electrolysis and the threshold function of aluminum (Al), manganese (Mn), and vanadium (V) precipitation. (**F**) Correlation between initial overpotential and the duration of stable electrolysis and the threshold function of Al, Mn, and V precipitation.

For quantitatively understanding the nonuniform geometric effects, in situ 4D characterization method was firstly used to study the geometric changes in the anodic processes, with purpose of establishing the dissolution principle of soluble anodes. As shown in Fig. 1A, a sponge titanium anode (processing into a cubic shape with size of 2 mm in side length and 5 mm in height) and a nickel wire cathode (cylinder shape with size of 1 mm in diameter and 5 mm in height) were used in a typical molten salt electrolysis (550°C, electrolyte: NaCl-LiCl-KCl salt). A constant current of a total current of 0.035A was applied to the electrolytic process (electrolysis time: 3 hours). Time-dependence 3D evolution behaviors of the entire soluble Ti anode structure (interval time at initial state, first hour, second hour, and third hour) were recorded via the in situ 4D characterization method (Fig. 1A). The nonuniform dissolution behaviors of the Ti anode could be acquired, which shows a fast dissolution rate at the bottom anode while a slow dissolution rate near the top of the molten salt.

To analyze the evolution of time-dependence nonuniform anodic dissolution at various electrode positions, the 3D reconstructed geometric images of Ti anodes were equational divided into five parts along the height direction, as shown in Fig. 1B. By analyzing the dissolution rate in different regions of the Ti anode, the quantitative height-dependence dissolution rate can be further established. Along the anode height (Fig. 1B), the height-dependence Ti dissolution rate and height-dependence current density were calculated by as-acquired the dissolution volume and surface area of each Ti anode section via AVIZO 2020. At each divided section of the Ti anode, the equation for calculating the time-dependence current density *j*_sec_(*x*,*t*) (*x* = 1, 2, 3, 4, and 5) is shown as follows

$$j_{\text{sec}}\left(x,t\right)=∆V_{\text{sec}}\left(x\right)\rho_{\text{Ti}}n/M_{\text{Ti}}FS_{\text{sec}-\text{anode}}\left(x\right)t$$ (1)

where $∆V_{\text{sec}}\left(x\right)$ is the dissolved volume of each titanium anode section (*x* = 1, 2, 3, 4, and 5), ρ_Ti_ is the density of the sponge Ti, *n* the number of charge transfer in the anodic reaction, *M*_Ti_ is the molar mass of Ti, *F* is the Faraday constant, $S_{\text{sec}-\text{anode}}\left(x\right)$ is the local electrode surface area of each divided anode (*x* = 1, 2, 3, 4, and 5), and *t* is the electrolytic time.

From the as-obtained current density $j_{\text{sec}}\left(x,t\right)$, the overpotential of different scanning time (*t* = 1, 2, and 3 hours) and volume section (*x* = 1, 2, 3, 4, and 5) can be calculated. Electrochemical dissolution of Ti is generally considered as a two-step reaction) (*2*)

$$\text{Ti}-2e^{-}↔{\text{Ti}}^{2+}$$ (2)

$${\text{Ti}}^{2+}-e^{-}↔{\text{Ti}}^{3+}$$ (3)

Equation 2 is considered as the rate-determining step. The overpotential of the divided Ti section at different scanning time can be obtained by Butler-Volmer equation of describing a multielectron transfer step

$$j\left(x,t\right)=i_{0}\left[e^{-\left(1+\alpha \right)f\eta \left(x,t\right)}-e^{\left(1-\alpha \right)f\eta \left(x,t\right)}\right]$$ (4)

where *i*_0_ is the exchange current density of the electrode, which can be measured by Tafel method (*2*). In this study, the experimentally measured exchange current density *i*_0_ of titanium electrode was 0.09A/cm^2^. *n* is the number of charge transfer in the reaction (*n* = 2). α represents the electrode reaction transfer coefficient. In most systems, α is in the range of 0.3 to 0.7, and it can be usually simplified or approximated as 0.5 in the absence of actual experimental values (*26*). Therefore, α ≈ 0.5 was simplified as the coefficient in the simulation. In the term of *f* = *F*/*RT*, *F* is the Faraday’s constant (96,500 C/mol), *R* is the gas constant [8.314 J/(mol·K)], *T* is the temperature (*T =* 823.15 K), and η(x,t) is the time-dependence overpotential at each divided anode (*x* = 1, 2, 3, 4, and 5).

Figure 1B shows the height-dependence evolution behaviors of Ti dissolution rate, current density, and overpotential. According to the fitted current density distribution data at different duration (0 to 1, 1 to 2, and 2 to 3 hours) (Fig. 1B), the current density increased from the anode near the molten salt top surface to the anode bottom. By using the Nernst Equation, the time-dependence current density $j_{\text{sec}}\left(x,t\right)$ (*x* = 1, 2, 3, 4, and 5) at different divided section could be rewritten as

$$j\left(x,t\right)=\mathrm{nF}\left[k_{f}\left(x,t\right)C_{\text{Ti}}\left(0,t\right)-k_{b}\left(x,t\right)C_{{\text{Ti}}^{2+}}\left(0,t\right)\right]$$ (5)

where $C_{\text{Ti}}\left(0,t\right)$ and $C_{{\text{Ti}}^{2+}}\left(0,t\right)$ represents the reactant (Ti) and product (Ti^2+^) concentrations on electrode surface, respectively. $C_{\text{Ti}}\left(0,t\right)$ = 1 is a constant. $k_{f}\left(x,t\right)$ and $k_{b}\left(x,t\right)$ represents the time-dependence reaction rate of Ti dissolution and deposition at different divided section (*x* = 1, 2, 3, 4, and 5) (Eq. 2), respectively, and can be expressed using Arrhenius formula

$$k_{f}\left(x,t\right)=A_{f}e^{-\frac{∆G_{\text{Af}}\left(x,t\right)}{\mathrm{RT}}}$$ (6)

$$k_{b}\left(x,t\right)=A_{b}e^{-\frac{∆G_{\text{Ab}}\left(x,t\right)}{\mathrm{RT}}}$$ (7)

where $∆G_{\text{Af}}\left(x,t\right)$ and $∆G_{\text{Ab}}\left(x,t\right)$ is the activation energy of forward and backward reactions of Eq. 2, *R* is the gas constant [8.314 J/(mol·K)], and *T* is the temperature (*T =* 823.15 K). In the reaction, the reactive solid-liquid interface varies along with electrolysis. Thus, it is assumed that each reaction site on the Ti surface will be accounted once, and this reactive site will disappear along with the Ti dissolution. $A_{f}$ and $A_{b}$ are frequency factors of forward and backward reactions of Eq. 2, both $∆G_{\text{Af}}\left(x,t\right)$ and $∆G_{\text{Ab}}\left(x,t\right)$ are solely dependent on the position *x* (i.e., the height direction of Ti anode) and could therefore be described as $∆G_{\text{Af}}\left(x\right)$ and $∆G_{\text{Ab}}\left(x\right)$ (*x* = 1, 2, 3, 4, and 5), respectively. To simplify the calculation, it is assumed that the mass transfer in the bulk electrolyte is a stable process. Thus, Ti^2+^ concentration is uniformly distributed in the bulk electrolyte, and it is assumed to nearly equal to that near the solid-liquid interface. Under these assumption conditions, Eq. 5 could be simplified as

$$j\left(x,t\right)=\mathrm{nF}A_{f}e^{-\frac{∆G_{\text{Af}}\left(x\right)}{\mathrm{RT}}}-{\mathrm{nFA}}_{b}e^{-\frac{∆G_{\text{Ab}}\left(x\right)}{\mathrm{RT}}}C_{{\text{Ti}}^{2+}}\left(t\right)$$ (8)

In this way, j(x,t) can be rewritten as a combination of three independent terms

$$j\left(x,t\right)=f_{1}\left(x\right)-f_{2}\left(x\right)f_{3}\left(t\right)$$ (9)

where the first two terms solely refer to the divided section *x* (*x* = 1, 2, 3, 4, and 5) of anode, and the third term solely refers to time *t*. These three terms could be expressed as

$$f_{1}\left(x\right)=\mathrm{nF}A_{f}e^{-\frac{∆G_{\text{Af}}\left(x\right)}{\mathrm{RT}}}$$ (10)

$$f_{2}\left(x\right)=\mathrm{nF}A_{b}e^{-\frac{∆G_{\text{Ab}}\left(x\right)}{\mathrm{RT}}}$$ (11)

$$f_{3}\left(t\right)=C_{{\text{Ti}}^{2+}}\left(t\right)$$ (12)

Equation 12 refers to the time-dependence concentration of Ti ions, which can be determined by calculating the dissolved volume of Ti anode under the assumption that the dissolved Ti is fully diffused into the bulk electrolyte. Note that Ti deposition on the Ni cathode is negligible because the electrolyte of low-concentration Ti ion was used to the in situ 4D characterization experiment (details in Materials and Methods). Therefore, $C_{{\text{Ti}}^{2+}}\left(t\right)$ could be described as

$$C_{{\text{Ti}}^{2+}}\left(t\right)=\frac{∆V_{\text{anode}}\rho_{\text{Ti}}}{\left(M_{\text{Ti}}N_{\text{salt}}+∆V_{\text{anode}}\rho_{\text{Ti}}\right)}=0.00682t$$ (13)

where $N_{\text{salt}}$ represents the molar amount of the molten salt (NaCl-LiCl-KCl salt) and $∆V_{\text{anode}}$ represents the anodic dissolved volume. According to the current density calculated by Eq. 1, the fitting result of Eq. 9 could be described as

$$j\left(x,t\right)=0.14\cdot e^{-0.6\cdot \left(x-3\right)}+20.5\cdot e^{0.6\cdot \left(x-3\right)}0.00682t$$ (14)

Combining with Eqs. 4, 9, and 14, the time-dependence overpotential η(x,t) and current density j(x,t) at different divided sections (*x* = 1, 2, 3, 4, and 5) could be expressed as

$$\begin{array}{c} j\left(x,t\right)=i_{0}\left[e^{\left(1-\alpha \right)\mathrm{nf}\eta \left(x,t\right)}-e^{-\left(1+\alpha \right)\mathrm{nf}\eta \left(x,t\right)}\right]=f_{1}\left(x\right)-f_{2}\left(x\right)f_{3}\left(t\right)\\ =0.14\cdot e^{-0.6\cdot \left(x-3\right)}+20.5\cdot e^{0.6\cdot \left(x-3\right)}0.00682t \end{array}$$ (15)

As a result, Fig. 1 (C and D) shows the relation of time-dependence current density j(x,t) and time-dependence overpotential η(x,t) at different divided sections (*x* = 1, 2, 3, 4, and 5), respectively. In each divided anode section, both the current density j(x,t) and overpotential η(x,t) are enlarged with increasing electrolysis time. At the bottom of Ti anode, the increment of both current density j(x,t) and overpotential η(x,t) is the largest due to the rapid dissolution caused by current accumulation at the edge of the electrode. At different divided sections (x = 1, 2, 3, 4, and 5), the dissolution rate of Ti anode is strongly linked with the electrode surface morphology. To achieve homogeneous dissolution of anode or deposition on the cathode, it is essential to uniformize the distribution of current density and overpotential on the solid-liquid interface.

To quantitatively evaluate the homogeneous dissolution of Ti anode, the threshold values of current density $j_{\text{cr}}$ and overpotential $\eta_{\text{cr}}$ were established. This threshold criteria of overpotential $\eta_{\text{cr}}$ (in the entire process) is determined by the overpotential of impurities $\eta_{\text{cr}\left(\text{impurity}\right)}$, as shown below

$$\eta \left(x,t\right)<\eta_{\text{cr}\left(\text{impurity}\right)}$$ (16)

In the Ti purification, three representative metals (Al, Mn, and V) were known as the impurities, and $\eta_{\text{cr}\left(\text{impurity}\right)}$ could be extended into three evaluation indexes [i.e., $\eta_{\text{cr}\left(\text{Al}\right)}$, $\eta_{\text{cr}\left(\text{Mn}\right)}$, and $\eta_{\text{cr}\left(V\right)}$ selected from (*27*–*28*)]. Note that general sponge titanium metal contains other impurities including Fe, Cr, Mo, Si, etc (*29*). As the dissolution potentials of Al, Mn, and V are most similar to Ti, selection of these three elements would best evaluate the optimization effect of the electrode design method established on the stability of the electrolytic process and product purity. These three threshold criteria $\eta_{\text{cr}\left(\text{Al}\right)}$, $\eta_{\text{cr}\left(\text{Mn}\right)}$, and $\eta_{\text{cr}\left(V\right)}$ could be used to plot the diagram of stable dissolution of Ti anode in the anodic process, as exhibited in Fig. 1E. To determine the criteria values, x = 5 was selected because the bottom anode presents the largest current density. Thus, $j_{\text{cr}}$ could be determined by j(5,t), as follows

$$j\left(5,t\right)<j_{\text{cr}}$$ (17)

Along with electrolysis time, the relation between $\eta_{\text{cr}\left(\text{impurity}\right)}$ and j(5,t) could be confirmed as follows

$$j\left(5,t\right)<j_{\text{cr}}=i_{0}\left[e^{\left(1-\alpha \right)\mathrm{nf}\eta_{\text{cr}\left(\text{impurity}\right)}}-e^{-\left(1+\alpha \right)\mathrm{nf}\eta_{\text{cr}\left(\text{impurity}\right)}}\right]$$ (18)

Therefore, the value of time-dependence η(5,t) should be smaller than $\eta_{\text{cr}\left(\text{impurity}\right)}$ (i.e., three indexes of $\eta_{\text{cr}\left(\text{Al}\right)}$, $\eta_{\text{cr}\left(\text{Mn}\right)}$, and $\eta_{\text{cr}\left(V\right)}$) according to inequation (*16*). Thus, the time-dependence threshold values and diagram for stable Ti dissolution versus applied current density could be determined, as shown in Fig. 1E. To further establish the link between η(5,t) and $\eta_{\text{cr}\left(\text{impurity}\right)}$, Eqs. 4 and 18 were combined to achieve the relation as below

$$\begin{array}{c} j\left(5,t\right)=i_{0}[e^{-\left(1+\alpha \right)\mathrm{nf}\eta \left(5,t\right)}-e^{\left(1-\alpha \right)\mathrm{nf}\eta \left(5,t\right)}]<\\ \left[e^{\left(1-\alpha \right)\mathrm{nf}\eta_{\text{cr}\left(\text{impurity}\right)}}-e^{-\left(1+\alpha \right)\mathrm{nf}\eta_{\text{cr}\left(\text{impurity}\right)}}\right] \end{array}$$ (19)

According to the above relation, the diagram for describing the stable Ti dissolution versus applied overpotential is plotted in Fig. 1F.

Figure 1 (E and F) shows the diagrams of threshold curves for current density and overpotential of different impurities, respectively. To achieve stable Ti dissolution in the electrolysis process, the applied current density (Fig. 1E) and the corresponding applied overpotential (Fig. 1F) for Ti dissolution cannot be higher than the threshold values of all the impurity dissolution (within the blue regions in both Fig. 1, E and F). In principle, the long-term dissolution of Ti anode would result in gradual decrease in the anode surface area and thus enhances the local current density on the anode interface, which in turn induces impurity dissolution in the anodic process. According to the established thresholds of different impurities, the applied current density on the whole anodes can be used to evaluate the anodic dissolution of impurities, and thus the stable anodic dissolution of Ti could be maintained. With consideration of the time-dependent nonuniform geometric effects, threshold values of the time-dependent applied current density (Fig. 1E) and time-dependent applied overpotential (Fig. 1F) could be used to evaluate the electrode design and applied processing parameters of Ti electrolysis, which would be discussed in the following sections.

### Nonuniform geometric effects of anodic process and evaluation of Ti dissolution

**Fig. 2.**
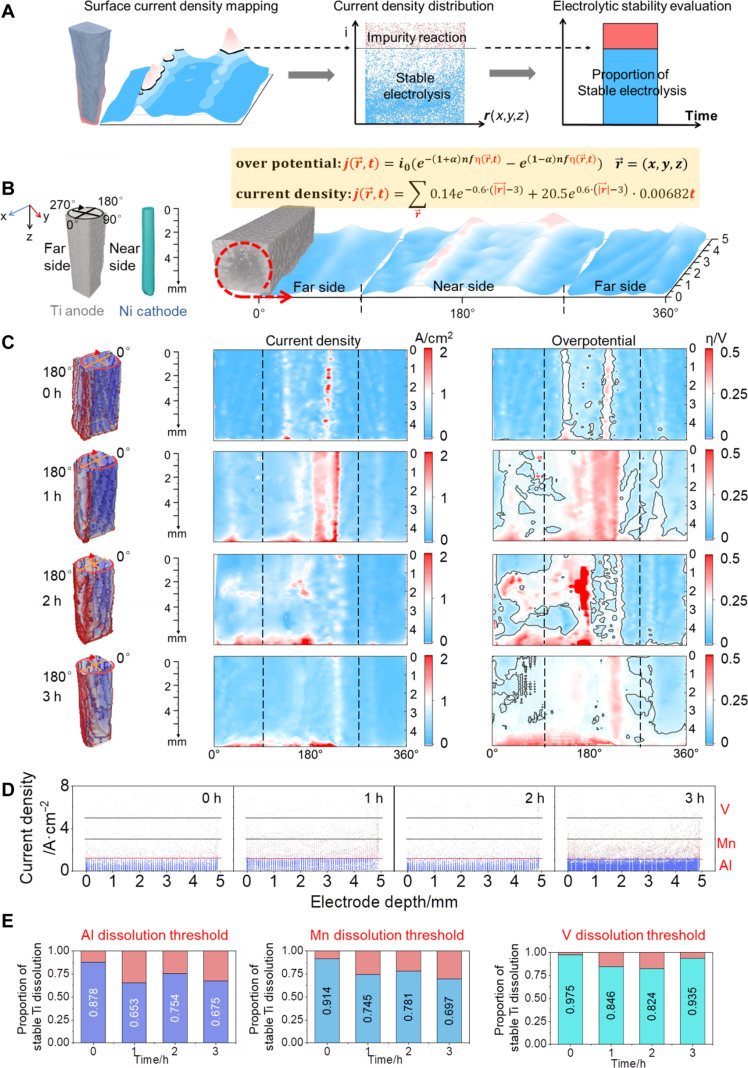
Nonuniform geometric effects of anodic process and evaluation of Ti dissolution. (**A**) Evaluation process of electrode current density/overpotential distribution. (**B**) Schematic of Ti anode surface development. (**C**) Evolution of Ti anode surface current density/overpotential simulation at different electrolysis time ranges. (**D**) Threshold segmentation of current density distribution at different electrolysis time ranges. (**E**) Evaluation of electrolytic stability based on precipitation of Al, Mn, and V.

**Fig. 3.**
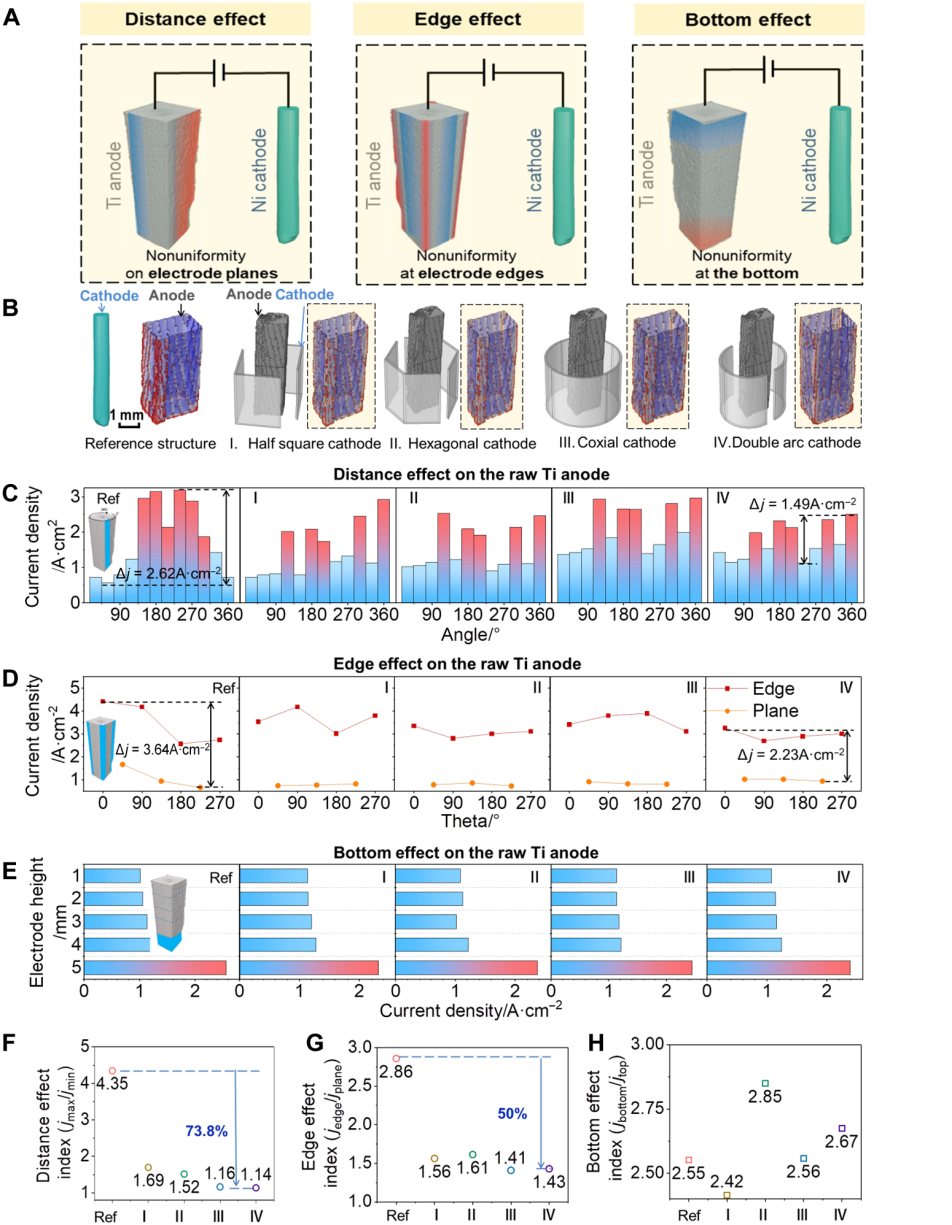
Cathode design configuration for uniform anode dissolution. (**A**) Three effects of nonuniform dissolution in anode. (**B**) Cathode design configuration and anode current density distribution in simulation. Scale bar, 1 mm. Geometric nonuniform current density distribution analysis on (**C**) distance effect, (**D**) edge effect, and (**E**) bottom effect. Indicator evaluation of (**F**) distance effect, (**G**) edge effect, and (**H**) bottom effect.

To well evaluate the stable Ti dissolution of the entire sponge Ti anodes, the 4D interface distribution of both applied current density [*j*(*x,y,z,t*)] and applied overpotential [η(*x,y,z,t*)] for Ti dissolution has been analyzed according to the in situ 4D experiments, where *x*, *y*, *z* refers to the spatial coordinate of anode surface, while *t* refers to the electrode electrolytic time (Fig. 2B). In a typical operation shown in Figs. 2A and 3D, the spatial surface current density of the sponge Ti anode with practical geometry was initially obtained by simulating the practical sponge Ti anode with the applied practical initial voltage in the COMSOL Multiphysics 5.6, followed by rolling the 3D distribution out into a 2D planar mapping. By introducing the diagrams of threshold curves for applied current density and applied overpotential (Fig. 1, E and F), the 2D areal proportion of current density distribution was determined via extracting the values higher than the threshold curves of all the impurities. The proportion of stable Ti dissolution was highlighted in blue region (Fig. 2A), while the proportion of impurity dissolution (Al, Mn, and V) was in the red region. To simplify the as-acquired distribution, the 3D spatial coordinate (*x,y,z*) was converted into the vector coordinate (*r*^→^), and thus *j*(*x,y,z,t*) and η(*x,y,z,t*) could be simplified as *j*(*r*^→^*,t*) and η(*r*^→^*,t*), respectively (Fig. 2B).

According to the above operation on the 4D experimental results, Fig. 2C exhibits the 2D planar mapping of the applied current density and applied overpotential of Ti dissolution. Along with Ti dissolution behaviors in Fig. 1A, the corresponding time-dependence distribution of the applied current density and applied overpotential has been well demonstrated. The blue regions show the proportion of stable Ti dissolution, while the red regions indicate the dissolution of impurities (Fig. 2C).

According to the analysis of 2D planar mapping, there are mainly three types of nonuniform distribution behaviors in the Ti dissolution. Type (1) refers to the geometric nonuniformity induced by variable anode-cathode distance (defined as the distance effect). As expected, the near region of Ti anode close to the cathode present higher values in applied current density and applied overpotential, which is linked with the greater electrical field intensity induced by the shorter distance. Along the electrolysis process, this distance effect would not be alleviated. Type (2) refers to the geometric nonuniformity induced by edge effects of the cubic electrode (defined as the edge effect). Initially, the values of the current density and overpotential presents higher at the edge regions of the cubic anodes, in comparison with the planar region of the anodes. Along the electrolysis process, the edge effect was found to be alleviated. Because the Ti dissolution rate at the edge regions was faster, the edge regions of the cubic anode would be decreased. Type (3) refers to the geometric nonuniformity induced by concentrated current density at the anode bottom (defined as the bottom effect). Compared with the distribution of applied current density and applied overpotential, increasing current density and overpotential have been observed at the bottom of the Ti anode, which is consistent with the phenomenon of geometric variation in Ti anode. Along the electrolysis process, the impurity dissolution (referred to the red regions) was prone to increase, and this bottom effect would be more pronounced, which is understood as the decreased surface area induced by the Ti dissolution.

To quantitatively calculate the regions of the applied current density higher than the threshold values of three impurities (threshold achieved in Fig. 1E), the distribution results of the applied current density for various electrolysis time are provided in Fig. 2D. Particularly, the regions lower than the current density required for Al precipitation (<0.6A·cm^2^) are shown in blue, and the other region (current density > 0.6A·cm^2^) is shown in red. As demonstrated in Fig. 2E, the dissolution process was separately evaluated on the basis of the dissolution threshold values of Al, Mn, and V (threshold achieved in Fig. 1E). The time-dependence quantitative proportions of stable Ti dissolution are provided in the blue regions of Fig. 2E. Along the electrolysis process, proportions of stable Ti dissolution would decrease. For example, the dissolution proportion of both Al and Mn impurities would be enlarged, with increment of ~20% (evaluated at the third hour) in comparison with the initial state.

According to the in situ 4D experiments and analysis, the quantitative evaluation criteria on the stable Ti dissolution and three types of geometric nonuniform distribution principle in the Ti dissolution have been achieved. These two criteria and principle would be used to design the structure configurations of cathodes and anodes for the molten salt electrorefining processes and quantitative evaluation of the uniformity and purity on the products.

### Cathode configuration design for uniform anode dissolution

According to the previous analysis on 4D experiments, the origin of the geometric nonuniformity dissolution of Ti anode can be divided into three reasons (Fig. 3A): (i) distance effect (distance variation between cathode and anode), (ii) edge effect, and (iii) bottom effect. To address the (i) distance effect, coaxial types of cathodes and anodes were used, including four types cathode configurations (I. half-square cathode, II. hexagonal cathode, III. coaxial cathode, and IV. double arc cathode) as shown in Fig. 3B. To verify improvement in the uniformity of current density distribution via the as-designed cathode configurations, the same anode structure (initial structure of the titanium sponge anode) was firstly used for simulation. Compared with the reference configuration (left cathode–right anode configuration in Fig. 3B), the anode current density distribution via the coaxial configuration design (type I to type IV in Fig. 3B) has been analyzed (fig. S7). According to the results, three types of the effects on nonuniformity dissolution of Ti anodes have been subsequently discussed.

To analyze the nonuniformity current density distribution caused by distance effect, the current density distribution on the anode surface was quantitatively plotted in Fig. 3C. In the typical operation, one side of the cathode was selected as the 0° on the *x*-*y* plane (Fig. 3B), 12 slices of the cathode surface (each 30° for one slice, from 0° to 360° along with *x*-*y* plane) were extracted to analyze the average current density on each selected slice. In the reference configuration (left cathode–right anode configuration), the anodic current density on the anode close to the cathode surface is higher than that away from the cathode surface, as exhibited in Fig. 3C. On the contrary, there is no evident geometric nonuniform distribution of anodic current density caused by the distance effect in the four coaxial electrode configurations (Fig. 3C). Particularly, the results demonstrate that the double arc cathode configuration (type IV. double arc cathode) exhibits a more uniform anodic current density among these five types of configurations (Fig. 3C).

To evaluate the alleviation of edge effect, the anodic current density distribution on the four edges of the raw square anode is further analyzed. In a typical operation, the anodic current densities on the four edges and three planes of each anode were selected to be plotted (Fig. 3D). Note that the fluctuation of the anodic current density on the double arc cathode configuration (type IV. double arc cathode) is in the range of 3.16 to 1.02 A cm^−2^, which is the smallest fluctuation range among all the cathode configuration (Fig. 3D). Thus, the edge effect could be substantially alleviated by the cathode configuration design. To analyze the bottom effect (Fig. 3E), on the other hand, there is no notable difference among these four coaxial electrode configurations in comparison with the reference configuration.

To further quantitatively evaluate the three types of effects on geometirc nonuniformity dissolution of Ti anodes, the corresponding three evaluation indexes were introduced in this study. These three indexes including ratio of the maximum to the minimum current density at different angles (*j*_max_*/j*_min_), ratio of the average current density of the four edges to total current density of the entire anode (*j*_edge_*/j*_plane_), and ratio of bottom current density to top current density (*j*_bottom_*/j*_top_) are used to evaluate the (i) distance effect, (ii) edge effect, and (iii) bottom effect, respectively. In the study of electrodeposition, only a few studies has quantitatively evaluated the 3D geometric uniformity of current density distribution using simulation (*30*). To emphasize the difference of geometric uniformity in various cathode configurations, we have carried out the evaluation method via the as-established three indexes in this study. Moreover, the comparison and discussion between the evaluation indexes are given in fig. S11.

Figure 3 (F to H) shows the evaluation index for three types of nonuniform geometric effects. The results demonstrate that the coaxial configuration design (types I to IV) would substantially reduce the distance effect and edge effect. According to the indexes, type IV presents decrease of 73.8 and 50% in distance effect and edge effect, respectively. However, the reduction on the bottom effect is limited, which could be further reduced via unique bottom structure design (discussed later).

### Anode configuration design and optimization for uniform deposition on cathode

**Fig. 4.**
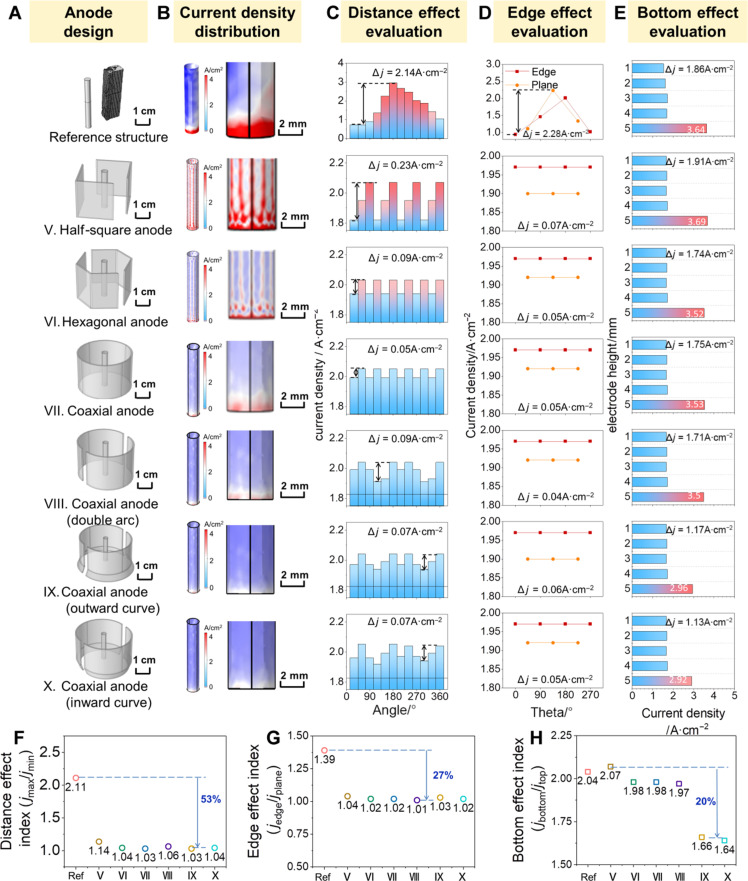
Anode configuration design and optimization for uniform deposition on cathode. (**A**) Anode design configuration (scale bars, 1 cm) and (**B**) cathode current density distribution (scale bars, 2 mm) in simulation, geometric nonuniform current density distribution analysis on (**C**) distance effect, (**D**) edge effect, and (**E**) bottom effect. Indicator evaluation of (**F**) distance effect, (**G**) edge effect, and (**H**) bottom effect.

On the basis of the understanding of the above electrode configuration design, the utilization of coaxial types of cathodes and anodes could eliminate the distance effect and edge effect. To meet the engineering conditions of practical electrorefining Ti, Ni cathode is positioned in the center, while the anode is coaxially positioned around the cathode, as shown in Fig. 4A (types V to X electrode configurations). The analysis method for Fig. 4 was based on the process of analyzing Fig. 3, with the purpose of promoting the uniformity of the cathodic deposition. In addition to the electrode configuration design of types V to VII (favorable for eliminating the distance effect and edge effect), electrode optimization on the cathode bottoms was applied to the coaxial anodes (types VIII to X electrode configurations), aiming at further alleviating the bottom effect. To quantitatively evaluate the three types of effects (i.e., distance effect, edge effect, and bottom effect on the Ti deposition on the cathode), four types of detailed analysis were carried out.

First, the cathodic current density distribution on the entire cathode/bottom is provided according to the simulated results, as shown in Fig. 4 (A and B). The simulation suggests that the anodes of coaxial configurations (types VII to X) present more current density distribution compared to the other two configurations (types V and VI). The type X electrode configuration exhibits the most uniform cathodic current density distribution.

Second, the quantitative analysis on the distance effect is provided in Fig. 4C. Likewise, the coaxial configurations (types VII to X) have more cathodic current density distribution at selected 12 slices around the cathodes in comparison with the other two configurations (types V and VI).

Third, the analysis on the edge effects was performed using the seven selected edges around cathodes, as plotted in Fig. 4D. The fluctuation of the cathodic current density on the type IV electrode configuration is in the range of 1.96 to 1.97 A cm^−2^, which could be considered the smallest fluctuation range among the six anode configurations.

Last, the bottom effects were quantitatively analyzed by evaluating the total cathodic current density on five sections along height direction. According to the column comparison shown in Fig. 4E, the cathodic current densities on the bottom section suggest that type X electrode configuration (coaxial configuration with an inward bottom) presents the smallest current density of 2.54 A cm^−2^ among all the electrode configurations.

Figure 4 (F to H) shows the evaluation indexes for three types of nonuniform geometric effects. Compared with the indexes of the reference configuration, the decreased values of the distance effect, edge effect, and bottom effect of type X electrode configuration (coaxial configuration with an inward bottom) are 53, 27, and 20%, respectively. Thus, type X electrode configuration is considered as the optimized design for anode-cathode configuration for stable Ti electrorefining, and the following experimental verification would be carried out.

### Experimental verification of anode-cathode configurations in Ti electrorefining

**Fig. 5.**
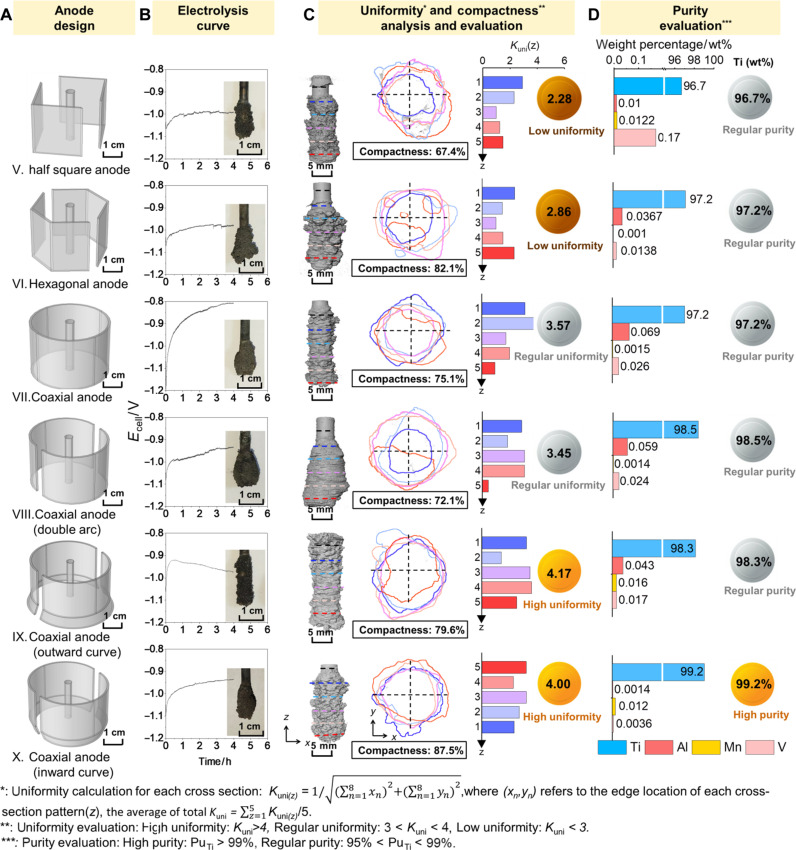
Experimental verification of anode-cathode configurations in Ti electrorefining. (**A**) Anode design configuration (scale bars, 1 cm). (**B**) Electrolysis curves and deposition products. Scale bars, 1 cm. (**C**) Uniformity and compactness evaluation (scale bars, 5 mm) and (**D**) purity evaluation for products.

To verify the rational design and optimization of the anode-cathode configurations based on the evaluation criteria and geometric nonuniform distribution principle, electrolysis experiments for Ti electrorefining were carried out using an electrolysis furnace (size: 150 mm in diameter, 800 mm in height). In a typical experiment, a cylindrical Ti rod with a diameter of 4 mm was used as the cathode (with the purpose of performing purity test without introducing impurities into the cathode matrix), and six different types of anode configurations (type V: half-square anode, type VI: hexagonal anode, and types VII to X: coaxial anodes with various optimized shapes in Fig. 5A) were fabricated according to the anode-cathode configuration design in Fig. 4A. In the experiment, the cathode current was calculated by electrolysis with constant current of 0.2A/cm^2^ for 4 hours. Furthermore, the distribution uniformity of Ti deposition products on the cathodes was characterized by CT imaging, followed by 3D reconstruction to verify the elimination of three geometric nonuniformity effects via anode-cathode configuration design in Fig. 4. In addition, the purity of the Ti deposition products was characterized by inductively coupled plasma (ICP).

The experimental distribution uniformity and purity of Ti deposition products for six types of anode configurations are demonstrated in Fig. 5 (C and D). To evaluate the distribution uniformity, the morphology analysis on 3D reconstruction was carried out via selecting five cross-section patterns of the Ti deposition products (equal distance from each selected section along the height). A parameter *K*_uni_ was introduced to evaluate the distribution uniformity for each cross-section pattern via the relation of *K*_uni(*z*)_*=*$1/\sqrt{{\left(\sum_{n=1}^{8}x_{n}\right)}^{2}+{\left(\sum_{n=1}^{8}y_{n}\right)}^{2}}$ (*n* = 8, *z* = 1, 2, 3, 4, and 5), where (*x_n_*,*y_n_*) refers to the edge location of cross-section pattern (Fig. 5C). The center of the cross-section pattern from each cylinder cathode was set as (0,0), and *K*_uni(*z*)_ of the total five cross-section patterns were calculated. As exhibited Fig. 5B, the columns present the *K*_uni(*z*)_, and the average of total *K*_uni(*z*)_ [i.e., *K*_uni_*=*$\sum_{z=1}^{5}K_{\text{uni}}\left(z\right)/5$] was determined as the values of distribution uniformity for each Ti deposition products. According to the standard of titanium purity, the product obtained by using type X electrode configuration meets the level of TA2 pure titanium (*31*). Therefore, the product purity obtained by using type X electrode configuration was defined as gold medal. Besides, the rest products with purity lower than 99 wt % (while higher than the anode purity) were defined as the silver medal. Noticeably, there is few report on quantitatively evaluating the uniformity of deposition products, and we have established the criteria via evaluating reconstructed 3D images of the products by CT (high uniformity (gold medal): *K*_uni_ > 4; regular uniformity (silver medal): 3 < *K*_uni_ < 4; low uniformity (copper medal): *K*_uni_ < 3). The corresponding values suggest that the Ti deposition products of types IX and X electrode configurations have the highest uniformity values of 4.17 and 4.00, respectively (Fig. 5C). On the other hand, the purity calibration from ICP demonstrates that the Ti deposition product using type X electrode configuration has the highest purity beyond 99% (defined as the high-purity level) compared to the other five types of anode configurations (96.7 to 98.3%, lower than the high-purity level) (Fig. 5D). In overall, the type X electrode configuration is evaluated to be the most optimized cathode-anode configuration among all the designs, which in turn confirms the validity of the as-proposed electrode design method.

In overall, the electrode design principle in this study (Figs. 4 and 5) is a universal method and can be extended to other electrochemical systems in the case that the uneven distribution of driving force is caused by the geometric effects of electrode shapes.

### Total evaluation of anode-cathode configurations in Ti electrorefining

**Fig. 6.**
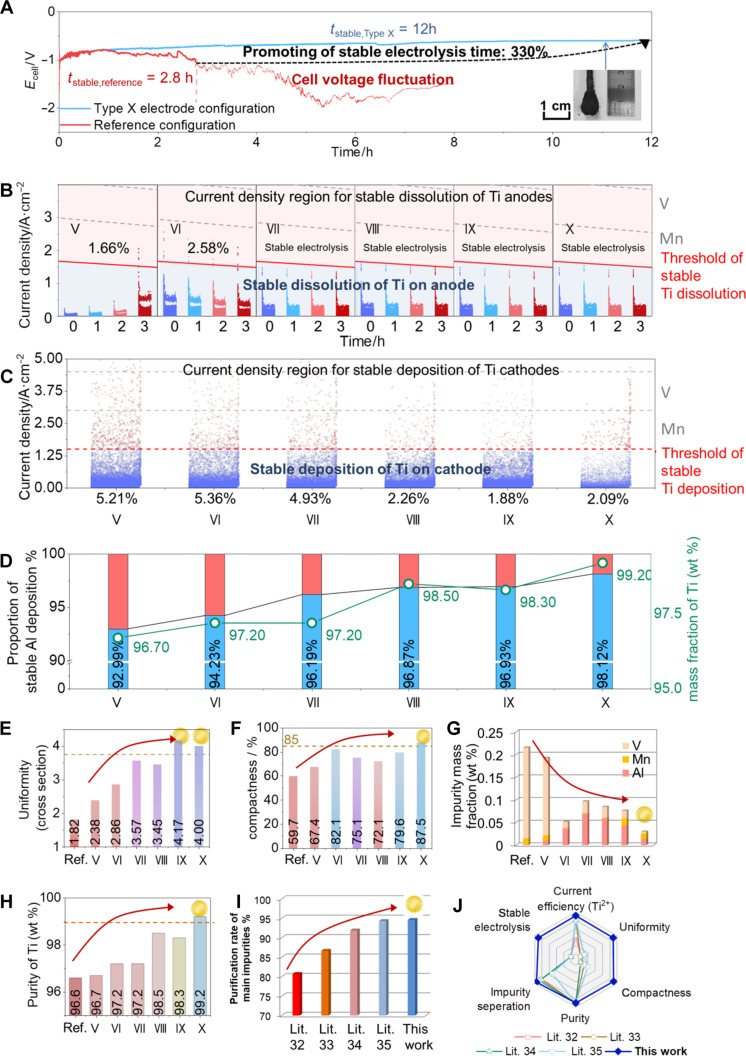
Total evaluation of anode-cathode configurations in Ti electrorefining. (**A**) Long-term Ti electrorefining up to 12 hours in the electrolytic furnace with stable voltage using type X electrode configuration (electrolysis stability: 330% increment in comparison with 2.8-hour stable electrorefining using the reference configuration) (scale bar, 1 cm). Threshold segmentation of current density distribution of different (**B**) anode design configuration and (**C**) cathode. (**D**) Proportion of stable Al deposition and product purity of different anode design configuration. Comparison between different anode designs of (**E**) uniformity, (**F**) compactness, (**G**) mass fraction of impurities, and (**H**) purity of Ti. Comparison between this work and literature of (**I**) purification rate of main impurities (Al, Mn, and V in this work) and (**J**) overall performance.

To evaluate the stability of long-term electrorefining in the electrolysis furnace (size: 150 mm in diameter, 800 mm in height), comparison of Ti electrorefining using type X electrode configuration and reference configuration was carried out, while the stable cell voltage curves were recorded. As shown in Fig. 6A, the Ti electrorefining using type X electrode configuration could remain stable cell voltage up to 12 hours, which is ~330% increment compared to the Ti electrorefining using the reference configuration (cell voltage for 2.8 hours). the results prove that the cathode-anode design of type X electrode configuration can massively extend the stable electrolysis time for Ti electrorefining.

To further evaluate the anode configuration designs from Fig. 4A, quantitative current density distribution on both anodes and cathodes was evaluated via simulation on the 3D anode-cathode configurations (fig. S9). According to the threshold values of applied anodic current density established in Fig. 1E, the corresponding proportions of stable Ti dissolution for each electrode configuration were obtained in Fig. 6B (with the same operation as Fig. 2A). Therefore, the time-dependent proportions could be used to evaluate the stability of anodic Ti dissolution of each electrode configuration. Apparently, all the coaxial anodes (types VII to X electrode configurations) remain stable Ti dissolution behaviors, while the other two types exhibit 1.66 and 2.58% proportion of impurity dissolution in types V and VI electrode configurations, respectively.

On the other hand, this method could also be used to evaluate the stability of cathodic Ti dissolution behaviors. As shown in Fig. 6C, the threshold of applied cathodic current density was established via selecting the initial value of Al threshold (*t* = 0) in Fig. 1E. Noticeably, the selection of the initial values for applied cathodic current densities is attributed to the factor that the theoretical anodic and cathodic overpotentials per surface unit on the of Ti anode and Ti cathode (used in the electrorefining experiment) are in the same absolute value (but positive for anodic overpotential and negative for anodic overpotential). Along with the electrolysis, the surface area of Ti anode is decreased, while the surface area of Ti cathode is increased. As a result, increasing anodic current density, i.e., decreasing threshold for impurity, could be obtained in Fig. 6B. On the contrary, decreasing cathodic current density, i.e., increasing threshold for impurity, could be achieved in Fig. 6C, which also implies that the initial value is the lowest threshold value. Therefore, the initial value of Al threshold (*t* = 0) in Fig. 1E could be selected as the applied cathodic current density in this study.

According to the proportions of stable Ti deposition on the Ti cathode (Fig. 6C), the cathodes matched with the optimized coaxial anodes (types XI to X electrode configurations) have larger stable proportions (~98%) than those with the other four types (95 to 97% in types V to VIII electrode configurations). Consequently, the results in proportions of stable Ti dissolution (Fig. 6A) and stable Ti deposition (Fig. 6C) indicates that the coaxial anodes (types XI to X electrode configurations) should be the ideal configurations for stable Ti electrorefining.

To verify the consistence of purity in Ti products (green circles in Fig. 6D) and proportion for stable Ti deposition (blue columns in Fig. 6D), the variation trend for these two factors has been compared, showing similar increase as exhibited in Fig. 6D.

To evaluate the quality of the as-deposited Ti products, uniformity, compactness, and mass fractions of impurities were demonstrated in Fig. 6 (E to G, respectively). The results suggest that the Ti products achieved using the Ti cathode matched with optimized coaxial anode (type X electrode configuration) present the highest total evaluation level among all the types. Compared to the reference configuration (Fig. 6, E to G), the increment of uniformity, compactness, and mass fractions of impurities in the Ti products achieved via type X electrode configuration was estimated to be 112, 39, and 95%, respectively.

Last, the purification rate in this work is compared to other electrolytic methods in the literature (Fig. 6I) (*32**–**35*). According to the comparison with the other in Fig. 6I, the as-designed coaxial anode-cathode configuration based on the strategy via in situ 4D characterization here presents substantial improvement in purification rate (up to 95%). Also, the as-designed type X electrode configuration has pronounced enhancement in total performance, including current efficiency, uniformity, compactness, purification rate, and product purity (Fig. 6J). As a consequence, the results in this study suggest that the design strategy could be used to remarkably enhance the stability of the electrorefining process and to promote the product quality in metallic refining.

## DISCUSSION

**Fig. 7.**
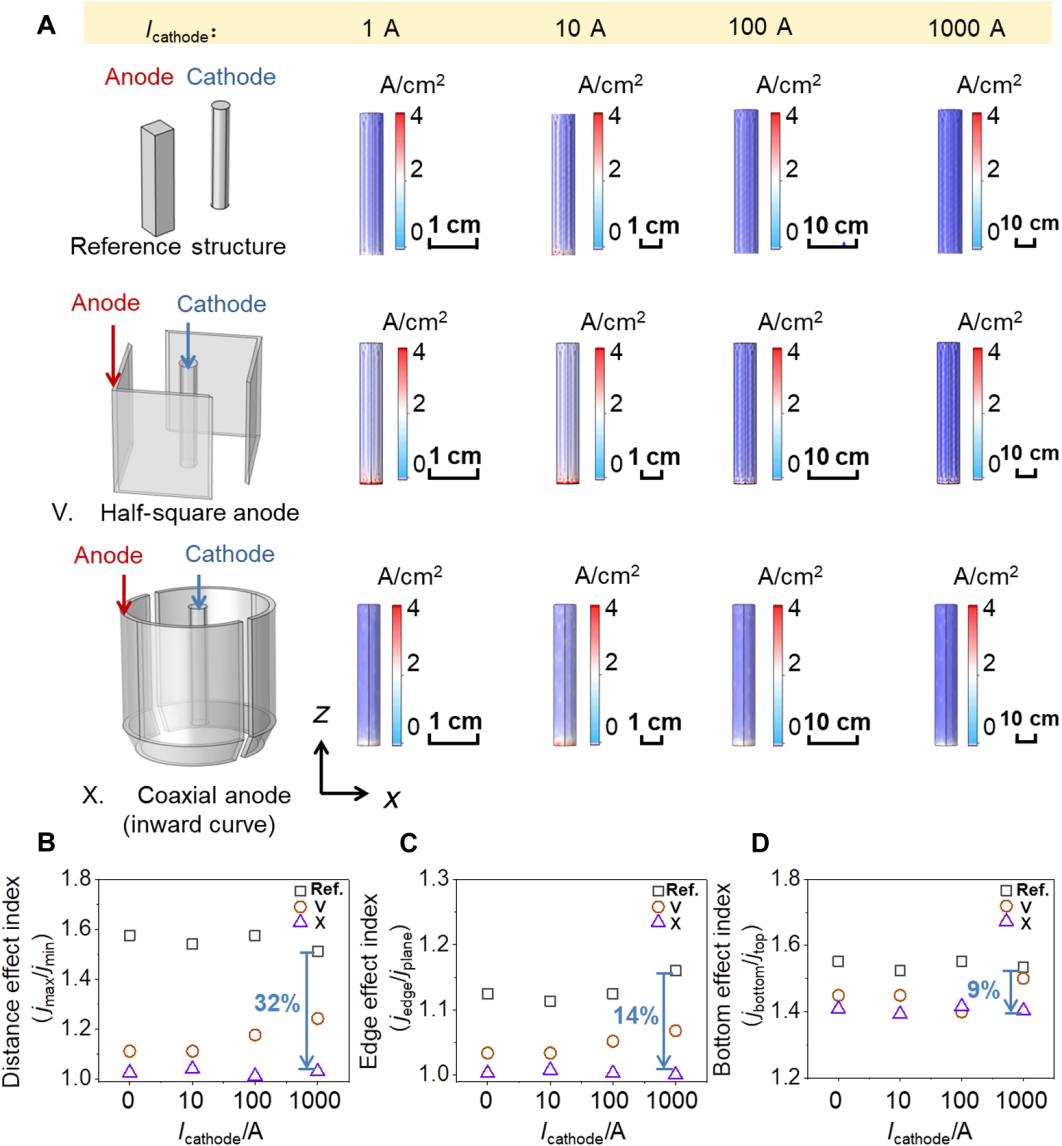
Uniformity comparison of current density distribution on the cathodes at different scales. (**A**) Uniformity comparison of current density distribution at different scales: Electrode configurations of reference structure, type V, and type X. (**B**) Distance effect indexes at different scales: reference structure, type V, and type X. (**C**) Edge effect indexes at different scales: reference structure, type V, and type X. (**D**) Bottom effect indexes at different scales: reference structure, type V, and type X.

To extend the validity of eliminating the nonuniform geometric effects via designing anode-cathode configurations, a simplified simulation was carried in the scale range of centimeter to meter (corresponding to the electrolysis conditions in the cathodic current range of 1 to 1000 A). It is noted that the condition of convection flow is not considered in the simulation. In a typical simulation, three representative electrode configurations, i.e., reference structure, type V, and type X, were selected. As exhibited in Fig. 7, the analyzed uniformity of current density distribution for each type of electrode configuration at different scales is provided. The results suggest that the three types of nonuniformity effects proposed in this work could be observed in the simulated scale range of centimeter to meter (Fig. 7A). The analysis also indicates that type X electrode configuration has the highest uniformity in the three indexes for nonuniform geometric effects. Compared to the reference structure, the values of the distance effect, edge effect, and bottom effect of type X electrode configuration at largest scale (1000 A) were decreased by 32, 14, and 9%, respectively (Fig. 7, B to D).

Therefore, the simplified simulation, in principle, suggests that the electrode design principle in Figs. 4 and 5 would be a universal method and can be extended to other electrolysis systems because the nonuniform geometric effects are commonly presented in the scale-up electrochemical systems and would affect the stability of long-term electrolysis. In practice, the analysis of nonuniform geometric effects would be considered in the electrode design; however, experimental validity should be carried out to optimize the electrode configurations and operation parameters to achieve the engineering goals.

In summary, this exploratory work provided a quantitative analysis method of electrolytic process based on high temperature in-situ electrochemical CT apparatus, and nonuniform geometric effects (i.e., distance effects, edge effect, and bottom effect) have been quantitatively discussed. To reduce the geometric nonuniformity of current density distribution caused by these three nonuniform geometric effects, four kinds of cathode configurations and six anode configurations were designed. Ultimately, the type X electrode configuration shows the most optimized uniformity in current density distribution, which has the highest uniformity (4.00) and purity (99.2 wt %) in the Ti product, along with a long-term stable electrorefining process for ~12 hours (~330% increment compared to reference configuration). Consequently, the strategy in the present study can be widely extended to other high-temperature electrochemical systems and industry, providing a pathway for achieving high-efficiency electrolytic process.

## MATERIALS AND METHODS

### In situ 4D experiment

The in situ 4D experiment was carried out on a high-temperature electrochemical computer microtomography (CT) platform (*2*). The in situ 4D facility is consist of a quartz tube electrolytic cell and a heating furnace with four halogen lamps (fig. S1). The constant-current electrolysis experiment remained 3 hours with a total current of 0.035A via CHI1140c electrochemical workstation on this platform. During the electrolysis, the entire electrolytic cell was scanned using the CT at each interval time, i.e., initial state, first hour, second hour, and third hour. The captured images from CT were transferred to the computer, and the image of electrodes was reconstructed into 3D images via AVIZO2020 and simplified via ABUQUS.

In the experiment, a sponge titanium (processed into a cubic shape with size of 2 and 5 mm) was used as an anode, and a nickel wire (cylinder shape with size of 1 mm in diameter and 5 mm in height) was used as a cathode. The sponge titanium anodes were obtained from Ningxia Deyun Chuangrun Titanium Co. Ltd, and the nickel wire cathode were purchased from Aladdin. The electrolyte [NaCl(8 mol %)–LiCl(55 mol %)–KCl(37 mol %) mixture salt] was purchased from Alfa Aesar.

### Experimental verification of anode–cathode configurations and characterization

The Ti cathode(>99 wt %) was obtained from Xinji Metal Material Co. Ltd, and the sponge titanium anode used in the experiment was obtained from Ningxia Deyun Chuangrun Titanium Co. Ltd and manually processed in the laboratory. The electrolysis experiment was carried out using vacuum atmosphere molten salt electroplating furnace NBD-G1200. The electrolyte of NaCl(6.3 mol %)–LiCl(53 mol %)–KCl(35.2 mol %)–TiCl*_x_*(4.5 mol %)(2 < *x* < 3) mixture salt was used in the experiment. The electrode height in molten salt is 2 cm. Note that types IX and X have an anode height in molten salt of 2.5 cm, with the anode higher than cathode for 0.5 cm. The applied current density is 0.2A/cm^2^, and the electrolysis time was set to 4 hours. The obtained Ti products were washed thoroughly using deionized water for three times and dried in vacuum oven at 80°C for 12 hours. Then, the Ti products were first characterized on the 4D facility and then tested via ICP, and the specific parameter was mentioned in table S2.

### Electrolysis simulation

The electrolysis simulations were carried out using ComsolMultiphysics 5.6. The physics model “tertiary current distribution and Nernst-Planck(tcd)” was used (*2*).

For the Ti dissolution and cathode configuration design section (Figs. 2 and 3), a two-electrode system was used in the simulation process; Ni is the cathode, and sponge titanium anode constructed above is the working electrode. The height of anode and cathode was set to 5 mm. The balance voltage distribution of cathode and anode was set to 0 and 2 V, respectively. The balance current of Ni cathodes was set to 0.04 A cm^−2^, and Ti anodes were set to 0.09 A cm^−2^. The diffusion coefficient of electrolyte ions was set to 2.72 × 10^−6^ cm^2^ s^−1^ in this molten salt electrolyte. The electrolyte concentration of Ti^2+^ was obtained using the data by experimental calculation (fig. S4). The temperature of the electrolytic cell was set to 773 K. The charge transfer coefficient of anode and cathode was set to 0.5 (table S1).

For the anode configuration design section (Figs. 4 and 7), both anode and cathode was set as titanium, and the balance current of Ti electrodes was set to 0.09 A cm^−2^. The balance voltage distribution of cathode and anode was set to 0 V. Other parameters are consistent with the above simulation.
