# Supplementary material for: Eliminating nonuniform geometric effects for long-term stable electrochemical extraction of high-purity titanium
Source: Sci Adv. 2025 Mar 21;11(12):eads7083. doi: 10.1126/sciadv.ads7083 (PMC11927662; doi:10.1126/sciadv.ads7083)
Supplement: Supplementary file 1 — Supplementary Text Figs. S1 to S11 Tables S1 and S2 [file sciadv.ads7083_sm.pdf]

Science Advances
AAAS

Supplementary Materials for

Eliminating nonuniform geometric effects for long-term stable electrochemical extraction of high-purity titanium

Zhiyuan Li *et al.*

Corresponding author: Shuqiang Jiao, sjiao@ustb.edu.cn; Zhaoliang Qu, quzl@bit.edu.cn; Wei-Li Song, weilis@bit.edu.cn; Dongbai Sun, sundongbai@mail.sysu.edu.cn

Sci. Adv. **11**, eads7083 (2025)
DOI: 10.1126/sciadv.ads7083

This PDF file includes:

Supplementary Text
Figs. S1 to S11
Tables S1 and S2

Supplementary Text

Derivation process of overpotential and current density expression

At each divided section of the Ti anode, the equation for calculating the time-dependance current density $j_{sec}(x,t)$ is shown as follows:

$$j_{sec}(x,t) = \Delta V_{sec}(x)\rho_{Ti}n/M_{Ti}FS_{sec-anode}(x)t, \quad \text{(S1)}$$

where $\Delta V_{sec}(x)$ is the dissolved volume of each titanium anode section (x=1, 2, 3, 4 and 5), ρ_{Ti} the density of the Ti, n the number of change transfer in the reaction, M_{Ti} the molar mass of Ti, F the Faraday constant, $S_{sec\text{-}anode}(x)$ the local electrode surface area of anode (x=1, 2, 3, 4 and 5), and t the electrolytic time.

$$Ti - 2e^- \rightarrow Ti^{2+} \quad \text{(S2)}$$

$$Ti^{2+} - e^- \rightarrow Ti^{3+} \quad \text{(S3)}$$

Eq. (2) is considered as the rate determining step. First, three hypotheses are introduced: 1. the whole reaction is regarded as a quasi-reversible process near equilibrium; 2. the electrolyte mass transfer is considered adequate; 3. the reaction interface is always in dynamic change, means that each reaction site is no longer an electrode surface site after participating in the reaction. For quasi-reversible process, the exchange current density of the rate-determine step could be expressed as:

$$i_{0,RDS} = nFk_{RDS}^0 C_{Ti^{2+}} e^{-\alpha f \eta_{RDS}} \quad \text{(S4)}$$

where $i_{0,RDS}$ represents the exchange current density of rate-determine step, k_{RDS}^0 the standard rate constant of rate-determine step, $C_{Ti^{2+}}$ the concentration of Ti^{2+}, α the electrode reaction transfer coefficient, and α=0.5 was selected for simplifying the calculation in this process. The term of $f=nF/RT$ is a constant, where n is the number of charge transfer in the reaction(n=2), F the Faraday's constant(96500 C/mol), R the gas constant(8.314 J/(mol•K)), T the temperature(T=823.15 K), and η_{RDS} the overpotential caused by rate-determine step. According to Nernst's equation in equilibrium:

$$e^{n' f \eta_{post}} = \frac{C_{Ti^{3+}}}{C_{Ti^{2+}}} \quad \text{(S5)}$$

where n' is the number of charge transfer in the post-reaction(n'=1), η_{post} the overpotential caused by post reaction(formula (3)), $C_{Ti^{3+}}$ the concentration of Ti^{3+}. Bring formula (5) into formula (4), the exchange current density of the rate-determine step could be expressed as:

$$i_{0,RDS} = nFk_{RDS}^0 C_{Ti^{3+}} e^{-\alpha f \eta_{RDS}} e^{n' f \eta_{post}} \quad \text{(S6)}$$

According to the BV equation, the current density of the total reaction can be expressed as:

$$j = -nF[k_f C_{Ti^{3+}}(0,\mathrm{t}) - k_b C_{Ti}(0,\mathrm{t})] \quad \text{(S7)}$$

$$k_{f'} = k_{RDS}^0 e^{-n' f \eta_{post}} e^{-\alpha f \eta_{RDS}} \quad \text{(S8)}$$

$$k_{b'} = k_{RDS}^0 e^{-(1-\alpha) f \eta_{RDS}} \quad \text{(S9)}$$

where $k_{f'}$ and $k_{b'}$ represent the reaction rate constant of the forward reaction and the reverse reaction, respectively. $C_{Ti^{3+}}(0,\mathrm{t})$ the concentration of Ti^{3+} at electrode surface. The division of equation (7) by equation (4) yields the following result:

$$\frac{j}{i_0} = \frac{C_{Ti^{3+}}(0,\mathrm{t})}{C_{Ti^{3+}}} e^{-(n'+\alpha) f \eta} + \frac{C_{Ti}(0,\mathrm{t})}{C_{Ti}} e^{(1-\alpha) f \eta} \quad \text{(S10)}$$

On the assumption, $C_{Ti^{3+}}(0,\mathrm{t}) = C_{Ti^{3+}}$, $C_{Ti}(0,\mathrm{t}) = C_{Ti} = 1$, therefore the end result is as follow:

$$j = i_0(e^{-(1+\alpha) f \eta} - e^{(1-\alpha) f \eta}) \quad \text{(S11)}$$

The relationship between current density and electrode height along with electrolysis time could be expressed as the following form:

$$j(x,t) = nF[k_f(x,t) C_{Ti}(0,t) - k_b(x,t) C_{Ti^{2+}}(0,t) \quad \text{(S12)}$$

where n is the number of charge transfer in the reaction(n=2), F the Faraday's constant(96500 C/mol), x represents the electrode height (x=1, 2, 3, 4 and 5), t represents the scanning time (t=1h, 2h and 3h). k_f and k_b represent the reaction rate of titanium dissolution and

deposition(Eq.(2)), respectively. $C_{Ti}(0,t)$ and $C_{Ti^{2+}}(0,t)$ represent the reactants(Ti) and product(Ti^{2+}) concentrations on electrode surface. Note that $C_{Ti}(0,t)$=1 as a constant. The reaction rate of titanium dissolution and deposition k_f and k_b can be calculated using Arrhenius formula:

$$k_f(x,t) = A_f e^{-\frac{E_{Af}(x,t)}{RT}} \tag{S13}$$

$$k_b(x,t) = A_b e^{-\frac{E_{Ab}(x,t)}{RT}} \tag{S14}$$

where A_f and A_b represents frequency factors of positive and reverse reaction of Eq.(2), $E_{Af}(x,t)$ and $E_{Ab}(x,t)$ the activation energy of positive and reverse reaction of Eq.(2), R the gas constant (8.314 J/(mol K)), T the temperature (T=823.15 K). Note that in the process of reaction, the reaction interface is always in dynamic change, means that each reaction site is no longer an electrode surface site after participating in the reaction. Therefore, E_{Af} and E_{Ab} can be regarded as solely dependent on spatial position x. To simplify the calculation, the electrolyte mass transfer is considered adequate, means that the bulk concentration of Ti^{2+} equals to the surface concentration and is evenly distributed in space. Thus, Eq. (5) could be expressed into the following form:

$$j(x,t) = nFA_f e^{-\frac{E_{Af}(x)}{RT}} - nFA_b e^{-\frac{E_{Ab}(x)}{RT}} C_{Ti^{2+}}(t) \tag{S15}$$

In this way, $j(x,t)$ can be regarded as a combination of three independent functions:

$$j(x,t) = f_1(x) - f_2(x) f_3(t) \tag{S16}$$

where

$$f_1(x) = nFA_f e^{-\frac{E_{Af}(x)}{RT}} \tag{S17}$$

$$f_2(x) = nFA_b e^{-\frac{E_{Ab}(x)}{RT}} \tag{S18}$$

$$f_3(t) = C_{Ti^{2+}}(t). \tag{S19}$$

The concentration of titanium ions, denoted as $f_3(t) = C_{Ti^{2+}}(t)$, can be determined by calculating the amount of anode dissolution under the assumption that the melt is fully transferred and there is negligible deposition on the cathode(The fitting result of $f_3(t)$ are shown in Figure S2):

$$C_{Ti^{2+}}(t) = \frac{\Delta V_{anode} \rho_{Ti}}{(M_{Ti} N_{salt} + \Delta V_{anode} \rho_{Ti})} = 0.00682t \tag{S20}$$

N_{salt} represents the molar amount of molten salt, ΔV_{anode} the anodic dessolution volume. According to the current density calculated by equation (1) above, the fitting results are as follows(The fitting result of $j(x) = f_1(x) - mf_2(x)$ are shown in **Figure S3**):

$$j(\boldsymbol{x},\boldsymbol{t}) = 0.14 \cdot e^{-0.6\cdot(\boldsymbol{x}-3)} + 20.5 \cdot e^{0.6\cdot(\boldsymbol{x}-3)} \cdot 0.00682\boldsymbol{t}, \tag{S21}$$

Through equation (4), the evolution law of overpotential $\eta(x,t)$ and current density $j(x,t)$ with time and space could be expressed as:

$$j(x,t) = i_0\left[e^{(1-\alpha)f\eta(x,t)} - e^{-(1+\alpha)f\eta(x,t)}\right], \tag{S22}$$

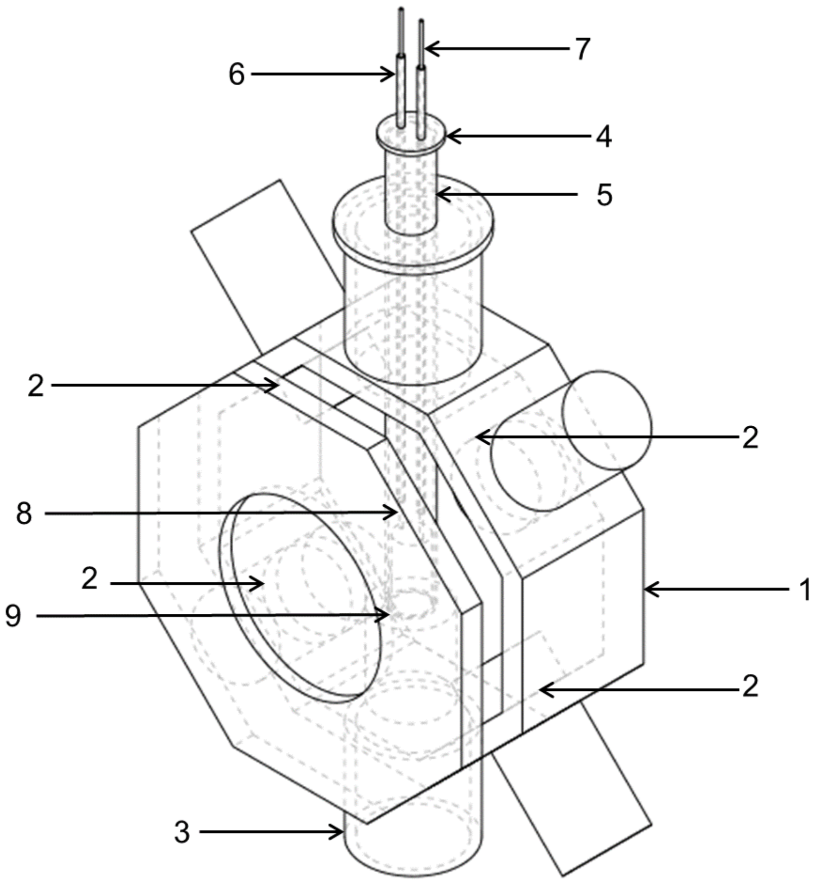

Figure S1 Structure of in-situ 4D facility. The composition is as follow: Aluminum-based alloy protection shell-1, Halogen lamp heating device-2, rotating base-3, crucible sealing cover (including holes)-4, quartz crucible-5, capillary quartz tube-6, electrode wire-7, crucible base-9

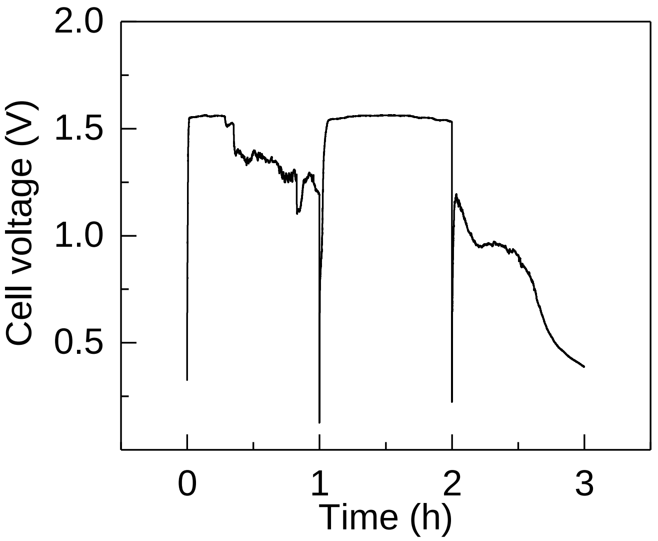

Figure S2 4D electrolytic test cell voltage curve. The 4D scan was carried out at the following time: t=0h, t=1h, t=2h and t=3h.

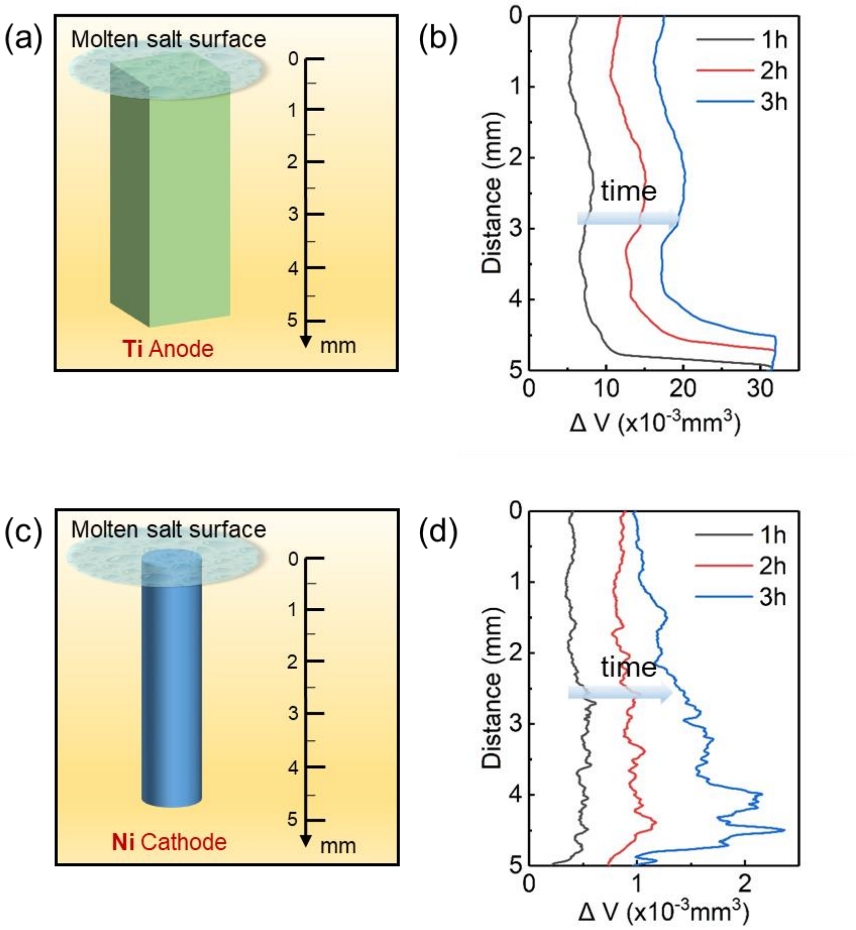

Figure S3 Volume change of anode and cathode along the electrode height. The schematic diagram of (a)Ti anode and (c) Ni cathode, and the volume change of (b)Ti anode and (d) Ni cathode along the electrode height.

Note that the volume change of sponge titanium anode exceeds that of the Ni cathode by an order of magnitude. Therefore, in the theoretical derivation presented in the manuscript, the titanium ion density in molten salt is directly calculated based on the dissolved quantity of titanium sponge anode.

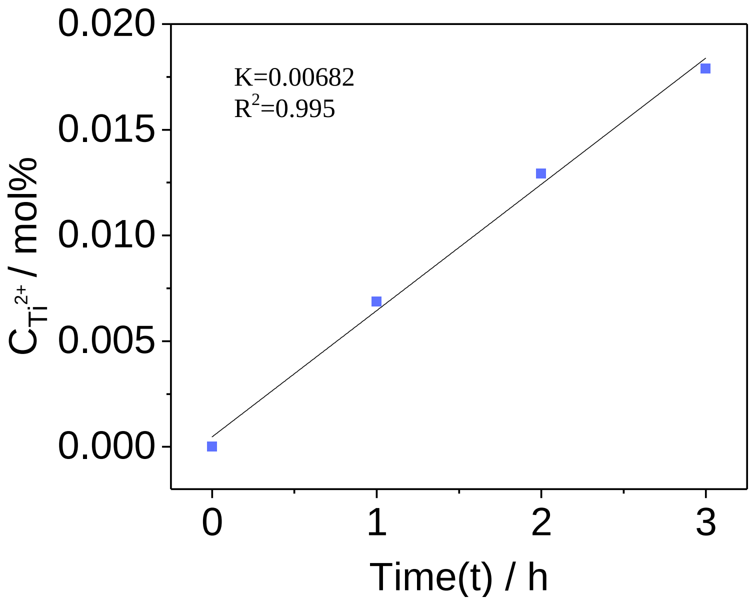

Figure S4 The fitting result of $f_3(t)$.

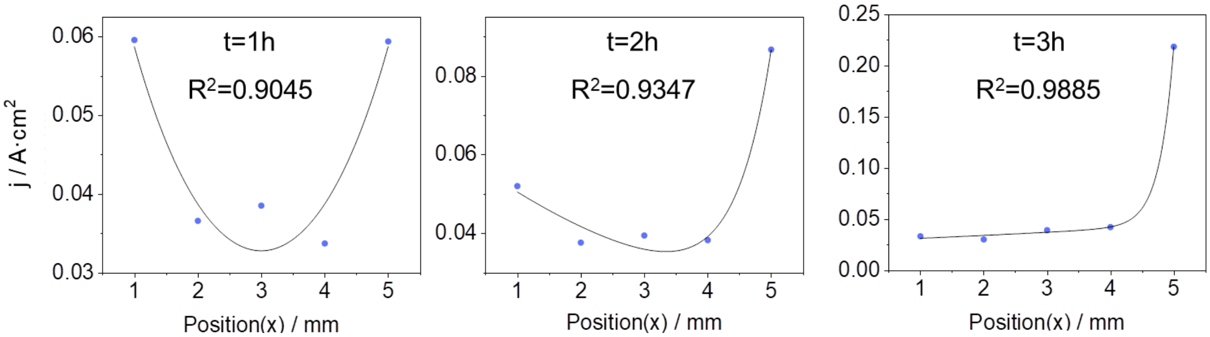

Figure S5 The fitting result of $j(x,t) = f_1(x) - f_2(x)f_3(t)$ with different *t* values.

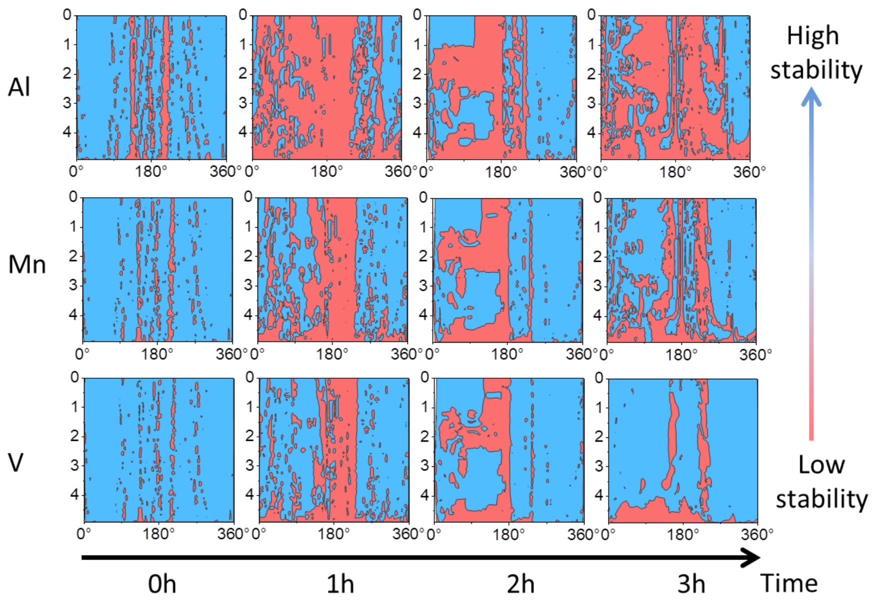

Figure S6 The 2D planar mapping of current density and overpotential of Ti anode with threshold of Al, Mn, V dissolution.

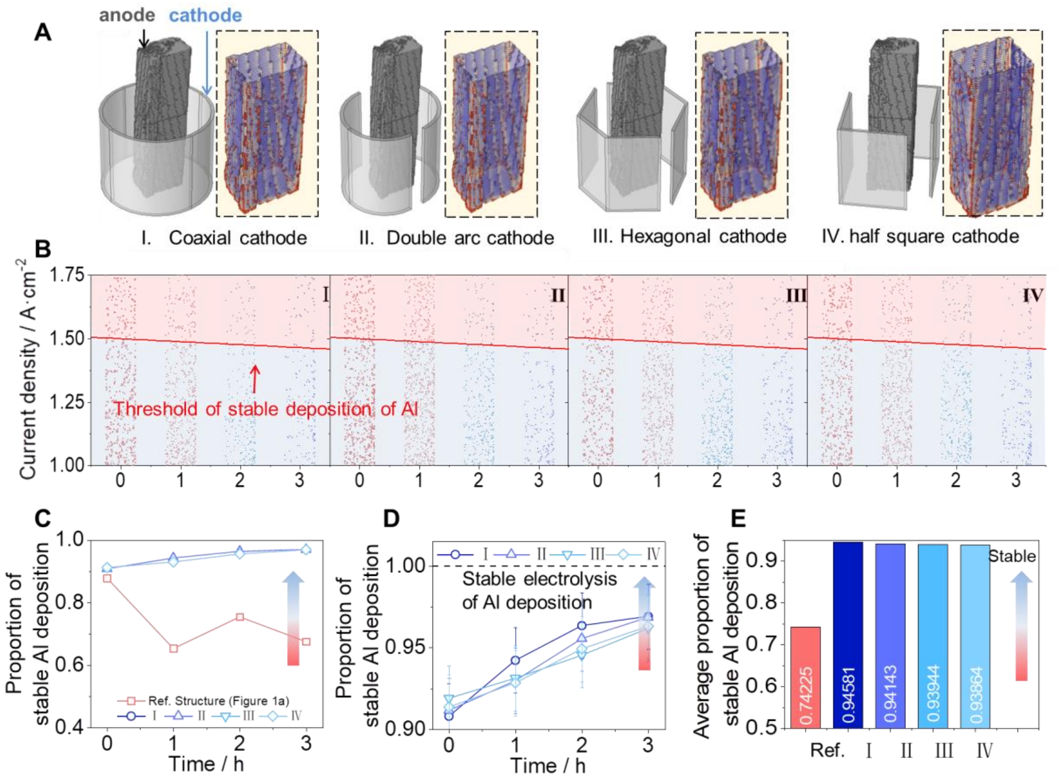

Figure S7 Cathode design configuration of uniform dissolution in anode. (**A**) Cathode design configuration and anode current density distribution in simulation. (**B**) Threshold segmentation of current density distribution of different cathode design configuration. (**C**-**D**) Proportion of stable Al deposition of refference electrode structure and different cathode design configuration. (**E**) Average proportion of stable Al deposition of refference electrode structure and different cathode design configuration.

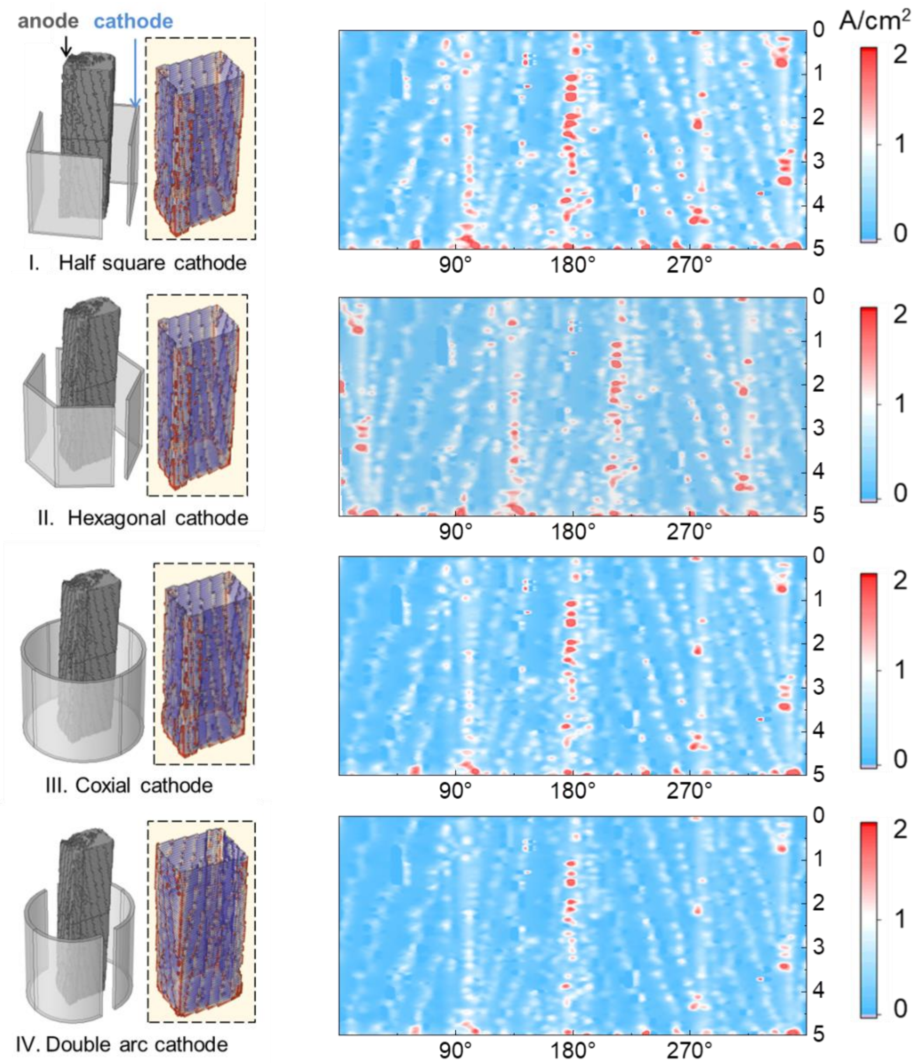

Figure S8 The 2D planar mapping of current density of Ti anode for cathode configuration design Type I to IV.

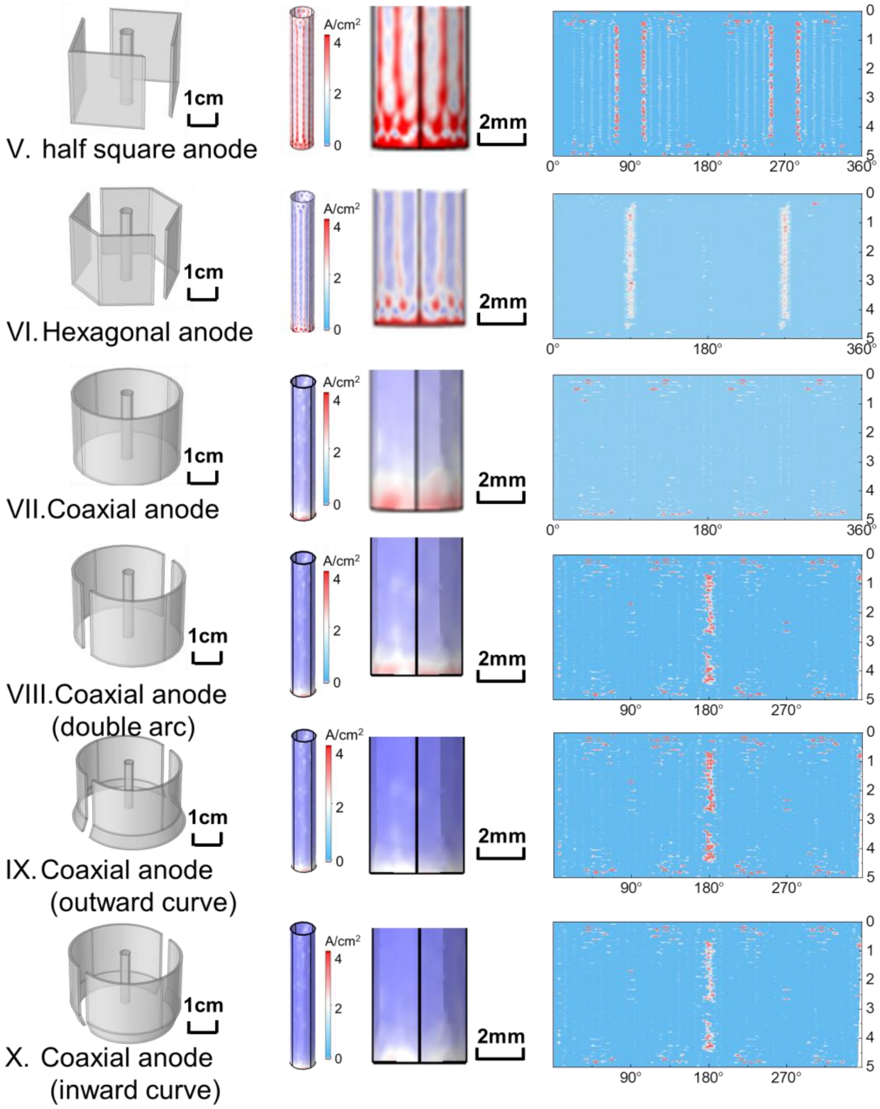

Figure S9 The 2D planar mapping of current density of Ti cathode for anode configuration design TypeV to X.

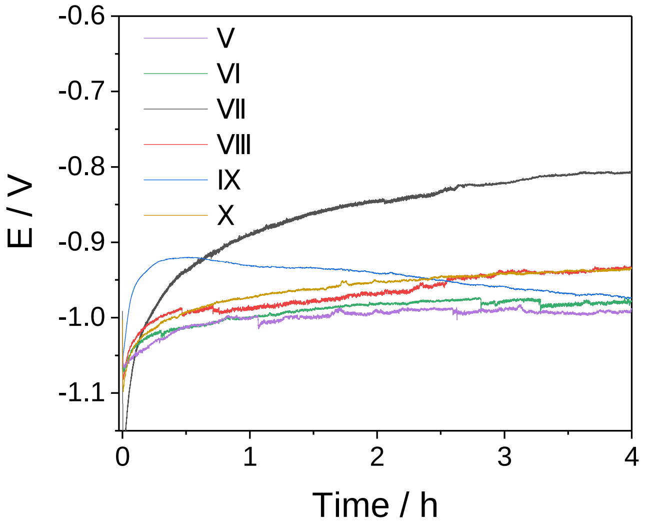

Figure S10 Cell voltage curves for anode configuration design TypeV to X.

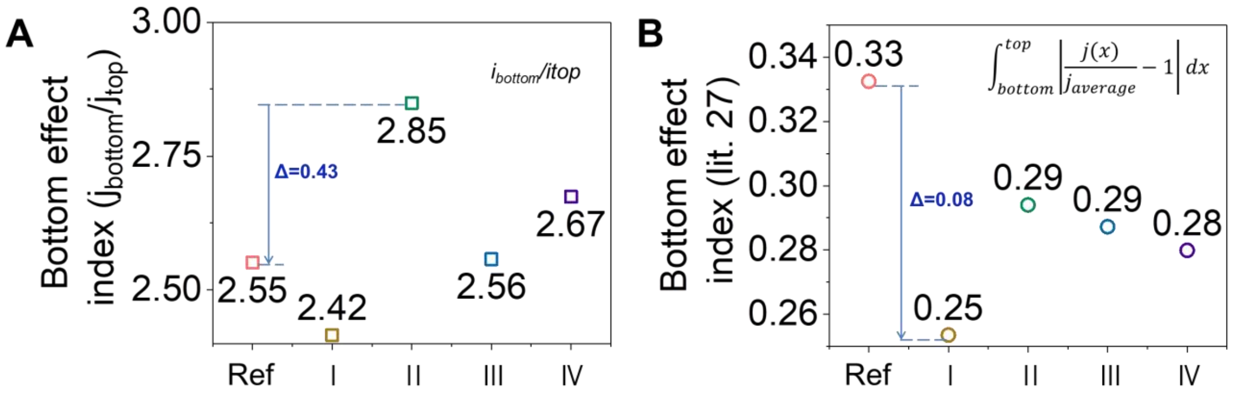

Figure S11 Bottom effect indexes using cathode configuration designs (Types I-IV). Comparison of bottom effect indexes between (**A**) this work and (**B**) previous study (*27*) using cathode configuration designs (Types I-IV).

In the study of the electrodeposition, only a few studies has quantitatively evaluated the three-dimensional geometric uniformity of current density distribution using simulation (*28*). In order to emphasize the difference of geometric uniformity in various cathode configurations, we have carried out the evaluation method via the as-established three indexes in this study. Moreover, the comparison and discussion between the evaluation indexes is given in Figure S11 in supplementary materials. As for bottom effct, the geometric non-uniformity has been documented in the literature as follow(*28*):

$$\text{geometric non-uniformity}=\int_{bottom}^{top}\left|\frac{j(x)}{j_{average}}-1\right|dx$$

where $j(x)$ refers the current density at electrode height x, $j_{average}$ refers to the average current density of the entire electrode. In view of this, we recalculated the bottom effect indexes of different electrodes according to this index, and compared it with the calculated results in this work. The result in **Figure S11** shows that both methods can reflect the non-uniformity of anode-cathode configurations along the radial direction, while the calculation method adopted in this work is more sensitive. Therefore, it can better reflect the change of bottom effect between different anode-cathode configurations. The above calculation results and discussion are given in manuscript and supplementary materials (**Figure S11** in supplementary materials).

Table S1 Parameter of COMSOL Simulation

Parameters	Values
Eeq_c (equilibrium potential of cathode)	0[V]
Eeq_a (equilibrium potential of anode)	2[V]
i0_c (balance current of cathode)	0.04[A/cm^2]
i0_a (balance current of anode)	0.09[A/cm^2]
be_c (charge transfer coefficient of cathode)	0.5
be_a (charge transfer coefficient of anode)	0.5
T (temperature)	773[K]
D (diffusion coefficient of electrolyte ions)	2.72×10^{-6}[cm^2/s]
rho_Ti (density of titanium)	4810[kg/m^3]

Table S2 Parameter of ICP-OES

Instrument Model	ICP-OES:Shimadzu ICPE-9800	
Instrument Parameter	Auto Sampler Speed	20r/min(Sample Flash)~60r/min(Solvent Flus)
	Carrier Flow	0.7L/min
	Auxillary Gas	0.6L/min
	Solvent Flush Time	30s
	Sample Flush Time	45s
	RF Power	1200w
